# Supplementary material for: Gelatin/Hyaluronic Acid Content in Hydrogels Obtained through Blue Light-Induced Gelation Affects Hydrogel Properties and Adipose Stem Cell Behaviors
Source: Biomolecules. 2019 Aug 5;9(8):342. doi: 10.3390/biom9080342 (PMC6722789; doi:10.3390/biom9080342)
Supplement: Supplementary file 1 [file biomolecules-09-00342-s001.pdf]

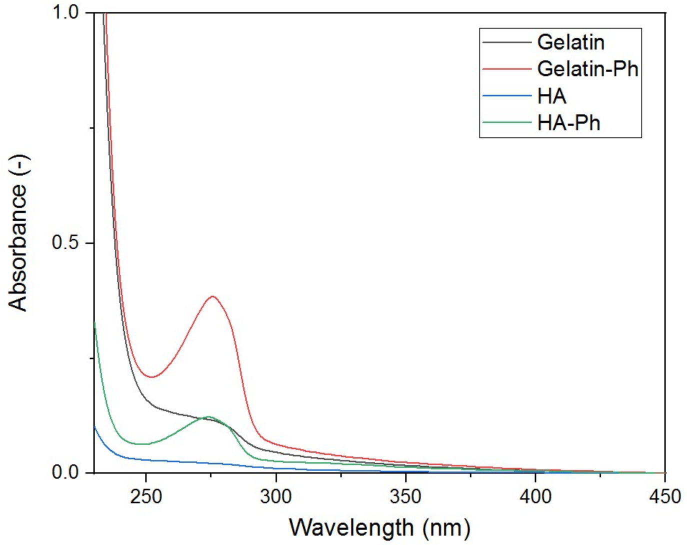

Supplemental Figure S1. UV-vis absorbance spectrum of unmodified gelatin, Gelatin-Ph, HA, and HA-Ph at 0.1 *w/w*%.

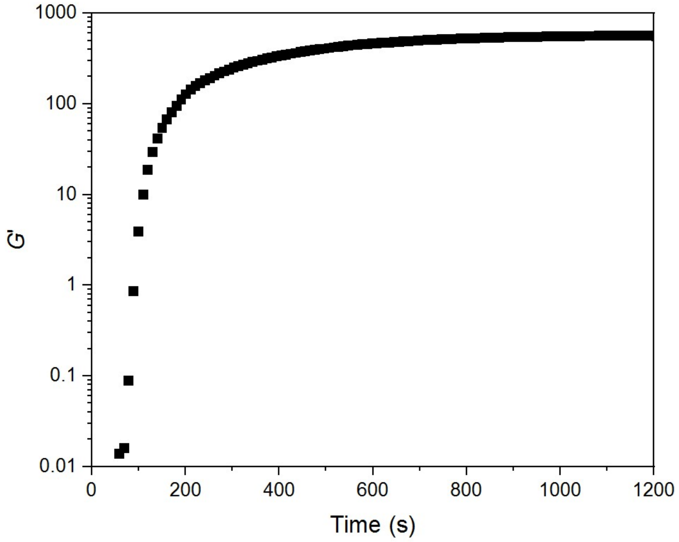

Supplemental Figure S2. Transition of storage elastic modulus of aqueous solution containing 1.0 *w*/*v*% alginate derivative possessing phenolic hydroxyl moieties, 1.0 mM $[Ru(bpy)_3]^{2+}$ and 1.0 mM SPS under blue light irradiation (33 mW/m^2 at 452 nm). The gelation time at this condition was 35 s.
